# Genetic background of juniper (*Juniperus spp.*) consumption predicted by fecal near-infrared spectroscopy in divergently selected goats raised in harsh rangeland environments

**DOI:** 10.1186/s12864-024-10009-7

**Published:** 2024-01-24

**Authors:** Henrique A. Mulim, John W. Walker, Daniel F. Waldron, Danilo G. Quadros, Lorena F. Benfica, Felipe E. de Carvalho, Luiz F. Brito

**Affiliations:** 1 Purdue University, West Lafayette, IN USA; 2 Texas A&M AgriLife Research and Extension Center, San Angelo, TX USA; 3 University of Arkansas System Division of Agriculture, Little Rock, AR USA; 4 São Paulo State University, Jaboticabal, São Paulo, Brazil; 5 Universtity of São Paulo, Pirassununga, São Paulo, Brazil

**Keywords:** Biological control, Genome wide association study, Monoterpene

## Abstract

**Background:**

Junipers (*Juniperus spp.*) are woody native, invasive plants that have caused encroachment problems in the U.S. western rangelands, decreasing forage productivity and biodiversity. A potential solution to this issue is using goats in targeted grazing programs. However, junipers, which grow in dry and harsh environmental conditions, use chemical defense mechanisms to deter herbivores. Therefore, genetically selecting goats for increased juniper consumption is of great interest for regenerative rangeland management. In this context, the primary objectives of this study were to: 1) estimate variance components and genetic parameters for predicted juniper consumption in divergently selected Angora (ANG) and composite Boer x Spanish (BS) goat populations grazing on Western U.S. rangelands; and 2) to identify genomic regions, candidate genes, and biological pathways associated with juniper consumption in these goat populations.

**Results:**

The average juniper consumption was 22.4% (± 18.7%) and 7.01% (± 12.1%) in the BS and ANG populations, respectively. The heritability estimates (realized heritability within parenthesis) for juniper consumption were 0.43 ± 0.02 (0.34 ± 0.06) and 0.19 ± 0.03 (0.13 ± 0.03) in BS and ANG, respectively, indicating that juniper consumption can be increased through genetic selection. The repeatability values of predicted juniper consumption were 0.45 for BS and 0.28 for ANG. A total of 571 significant SNP located within or close to 231 genes in BS, and 116 SNP related to 183 genes in ANG were identified based on the genome-wide association analyses. These genes are primarily associated with biological pathways and gene ontology terms related to olfactory receptors, intestinal absorption, and immunity response.

**Conclusions:**

These findings suggest that juniper consumption is a heritable trait of polygenic inheritance influenced by multiple genes of small effects. The genetic parameters calculated indicate that juniper consumption can be genetically improved in both goat populations.

**Supplementary Information:**

The online version contains supplementary material available at 10.1186/s12864-024-10009-7.

## Background

For more than a century, junipers (*Juniperus spp.*) have encroached on the Western U.S. rangelands, changing the landscape and reducing forage resources [1, 2]. These plants exert a significant negative influence on the structure and productivity of the plant community [3] as they compete with valuable forage plants for water and essential nutrients. Thus, it becomes crucial to implement effective juniper management practices to ensure the productivity of rangelands [4]. An efficient approach for addressing the juniper invasion lies at the intersection of science, ecology, and economics [5]. Various strategies can be employed to reduce or eliminate juniper from pastures, including the use of chemical herbicides [6], mechanical interventions [7], or prescribed fire [8]. However, the efficacy of these methods may vary depending on the juniper species, and some approaches may entail considerable costs and environmental damage. In this context, using goats (*Capra hircus*) as biological agents to control these invasive plants can be an efficient strategy [9].

Goats have an extraordinary ability to control invasive plants because of their grazing behavior [9]. Preference for juniper by browsing animals is negatively correlated to the concentration of plant secondary metabolites [10, 11]. When consumed in sufficient quantities, they can negatively affect intake [12], rumen microbial function, and cause hepatic injury [13]. Despite these challenges, goats have demonstrated a remarkable ability to consume chemically defended plants. This makes them the most promising livestock species for targeted grazing programs, compared to sheep and cattle, as goats appear to be less affected by the odor of the plants compared to other species [14].

In extensive systems, accurately estimating the total consumption of specific plants poses a challenge due to uncertainties in controlling what animals ingest. Fortunately, recent technological advancements offer promising solutions to bridge this gap of uncertain information. One such approach is the application of fecal near-infrared reflectance spectroscopy (fNIRS) to predict diet composition. Compared to standard laboratory procedures, fNIRS brings significant advantages, such as being non-labor-intensive, enabling rapid execution, treating samples non-destructively, and reducing chemical waste [15]. fNIRS enables the measurement of essential attributes in the diet selected by grazing herbivores, including crude protein content, coefficient of digestibility, botanical composition, and, in some cases, voluntary intake [16]. This proprietary method presents a unique opportunity to accurately estimate the consumption of specific plants. By leveraging fNIRS technology, valuable insights into animal foraging behavior can be obtained, which will improve the overall understanding of grazing ecology in extensive production systems.

Factors such as breed, sex, and animal age have been reported to influence juniper consumption by goats [12, 17]. Furthermore, there is evidence of individual variability, indicating that goats can be selected for increased juniper consumption [18]. However, there is limited research on the genetic background of this trait [19], including a lack of genomic-based variance components and identification of genomic regions and candidate genes associated with juniper consumption in goats. Therefore, the main objectives of this study were to: 1) calculate realized heritabilities and estimate variance components and genetic parameters, including heritability and repeatability, for predicted juniper consumption in divergently selected Angora (ANG) and composite Boer x Spanish (BS) goat populations grazing on Western U.S. rangelands; 2) investigate the relationship of juniper consumption prediction with a productive trait (i.e., weaning weight); 3) perform a genome-wide association study (GWAS) based on whole-genome sequence data (WGS); and 4) identify candidate genes associated with juniper consumption and the primary biological functions of these genes.

## Methods

### Animals and phenotypic information

The animals enrolled in this study were raised in herds managed by the Texas A&M AgriLife Research on typical juniper-infested rangeland in the Edwards Plateau of Texas, USA. All the procedures involving animals were approved by the Texas A&M University Institutional Agricultural Animal Care and Use Committee under protocols 2003–129 and 2018-021a. The predominant ecological site is a Low Stony Hill in an Oak/Mixed-brush Shortgrass seral state [20]. Woody plant canopy cover is about 35% and consists primarily of oak (*Quercus fusiformis*) and juniper (*Juniperus ashei* and *J. pinchotii*). Pricklypear (*Opuntia sp*.), algerita (*Mahonia trifoliata*), and other shrubby species are also common. Shortgrasses such as buffalograss (*Bouteloua dactyloides*), three-awns (*Aristida* sp.), and curlymesquite (*Hilaria belageri*), and the cool season Texas wintergrass (*Nassella leuctricha*) are also present in the pastures where the animals were raised. During the winter and long drought seasons, additional supplementation was provided to the goats based on an assessment of available forage quantity and quality. This supplementation encompassed either whole cottonseed or range cubes. Unlimited access to water and mineral mix was ensured.

Two goat populations were used for this study, including a Boer x Spanish (BS) composite population raised for meat production [21] and an Angora (ANG) population, which is a sample from a breed known for its fiber-producing attributes [21]. To assess juniper consumption, fecal samples were gathered through rectal palpation, followed by drying at 55 °C in a forced-air oven for 48 h. Subsequently, the samples were grounded using a cyclone mill (Cyclotec 1093, Foss, Hilleroed, Denmark). These ground samples were packed into quarter-cup sample cells equipped with a near-infrared transparent quartz cover glass. The sample cells underwent 32 scans using a scanning reflectance monochromator (model 6500, NIR Systems Inc., Silver Springs, MD, USA). The reflected energy (log[1/R]) was measured and averaged across 32 scans, recorded at 2-nm intervals spanning from 1,100 to 2,500 nm. The monochromator employing ISI NIRS2 version 3 software (Infrasoft International, Port Matilda, PA, USA) was used for the analyses. This setup enabled the collection of spectra and prediction of the juniper percentage in the diets using a Partial Least Squares equation previously developed. The predictive equation had an *r*^2^ value of 0.88, with a cross-validation standard error of 6.4% [17, 22]. Juniper consumption is usually higher in the dormant season, but the mean consumption over a 2-year period was highly correlated with spring samples [23]. In addition to seasonal variations in juniper consumption, there are also 7–9-day periodicities in juniper consumption [23]. Thus, the phenotypic estimate of the percentage of junipers in the diet was determined by sampling in the winter and spring seasons and collecting two samples three or four days apart in each season. When this study began, all goats were sampled each year. However, due to logistical constraints, sampling was reduced to four seasonal fecal collections (eight total collections) beginning with the first winter season after weaning. Both populations were raised on different ranches located in different counties, with varying plant populations and environmental conditions. Therefore, we avoided making direct statistical comparisons between them.

A total of 2,048 BS (1,161 females and 887 males) and 1,480 ANG (1,026 females and 484 males) goats were included in the study. The BS goats had an average (standard deviation) of 3.15 (± 1.33) juniper consumption records measured at an average age of 1.53 (± 0.67) years. The ANG animals had an average of 2.77 (± 1.64) phenotypic records measured at an average age of 2.02 (± 0.83) years. A comprehensive breakdown of the record count per animal is presented in Table 1. The pedigree datasets included 2,427 (BS) and 1,594 (ANG) animals with individuals recorded up the 8th (average: 4.44 ± 1.32) and 6th (average: 2.35 ± 1.66) generation, respectively. The average pedigree inbreeding was 0.04 and 0.01 for BS and ANG, respectively. The data generated was edited to remove inconsistent or outlier records for further analyses. The final dataset included 6,457 and 4,076 records from BS and ANG animals, respectively.

**Table 1:** Number of fecal samples per goat used for near-infrared spectroscopy determination of the percentage of juniper in the diets

	**Boer x Spanish composite**	**Angora**
**Number of samples**	**Number of animals**	**Number of animals**
1	111	372
2	752	461
3	188	193
4	885	303
5	15	50
6	41	89
7	19	21
8	15	18
9	8	2
10	4	1

Since 2003 and continued for over than 15 years, both goat populations were divergently selected to form HIGH and LOW selection lines, in which the HIGH line was selected to increase the percentage of juniper in the diet while the LOW line was selected to decrease the percentage of juniper in the diet. This was done to maximize the difference between divergent lines to improve our ability to identify physiologic and genetic differences between them.

### Genomic datasets

Genomic data from 501 BS and 210 ANG goats were obtained using the NovaSeq 6000 platform at a coverage depth of ~ 1X. The initial dataset comprised information of 407,644 autosomal SNPs. The alignment to the reference genome ARS1 [24] was performed using SAMTools v1.19 [25] while sorting, realignment, and quality filtering were managed with PICARD v1.16 [26]. For imputation, Beagle V4.0 [27] was employed, applying a Genotype Probability (GP) threshold of > = 0.90. After genotype imputation, 14,094,102 SNPs were obtained and used for the GWAS analyses.

A total of 74,199 SNPs were used for estimating variance components and genetic parameters. These SNPs were selected through a filtering process from the original dataset of 407,644 SNPs, excluding those with less than 20% of missing information and MAF < 0.05. Principal component analysis was conducted on the genotype information using PLINK v1.09 software [28]. As the PCA outcomes revealed two clearly distinct populations, all subsequent analyses were performed independently for both populations (BS and ANG).

### Variance components and genetic parameters

Variance components and genetic parameters were estimated for each goat population using genomic data from 501 (BS) and 210 (ANG) goats and 74,199 SNPs (as described above). The quality control process also involved evaluating the call rate for individual and genotypes (< 0.90) and identifying SNPs with an extreme departure from Hardy–Weinberg equilibrium (HWE *p*-value < 10^–10^). As a result, we obtained 40,187 and 50,957 informative SNPs for the BS and ANG populations, respectively.

The variance components were estimated based on Bayesian methods, using the BLUPF90 + family programs (GIBBS, POSTGIBBSF90) [29]. For the analyses, 1,000,000 cycles with a burn-in of 100,000 cycles and thinning of 100 samples were used. The “boa” R package [30] was used to evaluate convergence and posterior inference of the results. The single-step GBLUP approach (ssGBLUP) [31] was used to solve the mixed model equations. This method replaces the pedigree relationship matrix, combining pedigree and genomic relationship [32], as shown below:

$${\mathbf{H}}^{-1}={\mathbf{A}}^{-1}+\left[\begin{array}{cc}0& 0\\ 0& {\mathbf{G}}^{-1}- {\mathbf{A}}_{ 22}^{-1}\end{array}\right]$$

where **A** is the pedigree-based relationship matrix for all individuals in the population, **A**_22_ is the pedigree-based relationship matrix of the genotyped animals, and **G** is the genomic relationship matrix calculated as [33]:

$$\bf G=ZZ'$$

where **Z** represents a matrix containing adjustments for allelic frequencies. These adjustment factors were incorporated to align the mean diagonal of matrix **G** with **A**_**22**_, ensuring a close correspondence between the two matrices [34].

The following single-trait repeatability animal model was fitted:

$$\mathbf{y}=\mathbf{X}\mathbf{b}+\mathbf{Z}\mathbf{a}+\mathbf{W}\mathbf{p}\mathbf{e}+\mathbf{e}$$

where **y** is the vector of phenotypic observations (predicted juniper consumption); **b** is the vector of systematic effects representing the contemporary group defined based on the date of fecal collection (month and year), sex (male and female), and age in years; **a** is the random vector of additive genetic effects; **pe** is the random vector of permanent environmental effects; **e** is the random vector of residual errors; **X**, **Z**, and **W** are the incidence matrices associated with the systematic, additive genetic, and permanent environmental effects, respectively.

Contemporary groups with fewer than five individuals and records that deviated 3.5 standard deviations above or below the mean were excluded from further analyses. Residuals and permanent environment effects were considered to be independent and follow a normal distribution, characterized by a mean of zero and variances of **I**σ^2^_e_ and **I**σ^2^_pe_, respectively. The additive genetic effects were similarly assumed to adhere to a normal distribution, with a mean of 0 and a variance of **H**σ^2^_a_.

From the variance components obtained, the narrow sense heritability (h^2^) and repeatability (t; for juniper consumption) estimates were calculated as:

$$\begin{array}{ccc}{h}^{2}& =& \frac{{\sigma }_{a}^{2}}{{\sigma }_{a}^{2}+{\sigma }_{pe}^{2}+{\sigma }_{e}^{2}}\\ t& =& \frac{{\sigma }_{a}^{2}+{\sigma }_{pe}^{2}}{{\sigma }_{a}^{2}+{\sigma }_{pe}^{2}+{\sigma }_{e}^{2}}\end{array}$$

Realized heritability estimates were also calculated based on the Thompson’s approach [35].

### Genetic correlation with weaning weight

The analyses incorporated information on weaning weight from a total of 1,569 animals (805 females and 764 males) within the BS population, all born between 2006 and 2017. The animals were approximately 3.34 months old (with a standard deviation of ± 0.53 months) at the time of weaning weight measurement. Moreover, for the ANG population, the study included 598 animals (299 females and 299 males) born between 2009 and 2017. These animals were about 4.00 months old (with a standard deviation of ± 0.99 months) during the weaning weight assessment. Remarkably, 93.8% and 70.4% of the BS and ANG animals, respectively, had recorded information regarding juniper consumption. For further details on the descriptive statistics of weaning weight, please refer to Additional file 1: Table S1.

The genetic correlation with weaning weight was calculated based on bivariate analyses using the Bayesian methods implemented in the BLUPF90 + family software [21]. The following model was applied:

$$\bf \left[\begin{array}{c}{{\varvec{y}}}_{1}\\ {{\varvec{y}}}_{2}\end{array}\right]=\left[\begin{array}{cc}{{\varvec{X}}}_{1}& 0\\ 0& {{\varvec{X}}}_{2}\end{array}\right]\left[\begin{array}{c}{{\varvec{b}}}_{1}\\ {{\varvec{b}}}_{2}\end{array}\right]+\left[\begin{array}{cc}{{\varvec{Z}}{\varvec{a}}}_{1}& 0\\ 0& {{\varvec{Z}}{\varvec{a}}}_{2}\end{array}\right]\left[\begin{array}{c}{{\varvec{a}}}_{1}\\ {{\varvec{a}}}_{2}\end{array}\right]+\left[\begin{array}{cc}0& 0\\ 0& {{\varvec{Z}}}_{{\varvec{m}}2}\end{array}\right]\left[\begin{array}{c}0\\ {{\varvec{m}}}_{2}\end{array}\right]+\left[\begin{array}{cc}{{\varvec{W}}}_{1}& 0\\ 0& 0\end{array}\right]\left[\begin{array}{c}{{\varvec{p}}{\varvec{e}}}_{1}\\ 0\end{array}\right]+\left[\begin{array}{c}{{\varvec{e}}}_{1}\\ {{\varvec{e}}}_{2}\end{array}\right]$$

where **y**_**1**_ and **y**_**2**_ are the vectors of phenotypic observations for juniper consumption and weaning weight respectively; **b**_**1**_ is the vector of systematic effects for juniper consumption representing the contemporary group defined based on the date of collection (month and year), sex (male and female), and age in years; **b**_**2**_ is the vector of systematic effects for weaning weight representing the contemporary group defined based the year of birth and sex (male and female), the type of birth, and age in months as covariable; **a**_**1**_ and **a**_**2**_ are the vectors of direct additive genetic effects for juniper consumption and weaning weight respectively; **m**_**2**_ is the maternal genetic effects for weaning weight; **pe**_**1**_ is the vector of permanent environmental effects of juniper consumption; **e**_**1**_ and **e**_**2**_ is the vector of residual errors; **X**, **Za**, **Zm**, and **W** are the incidence matrices associated with the systematic, additive genetic, maternal genetic, and permanent environmental effects, respectively. The residuals and permanent environment effects were assumed to be independent and follow a normal distribution, characterized by a mean of zero and variances of **I**σ^2^_e_ and **I**σ^2^_pe_, respectively. The additive genetic and maternal genetic effects were similarly assumed to adhere to a normal distribution, with a mean of 0 and a variance of **H**σ^2^_a_ and **G**σ^2^_m_, respectively.

### Genome-wide association studies

A total of 14,094,102 SNPs were used for the genome-wide association studies (GWAS). Following the quality control described above, 5,309,518 and 8,052,245 SNPs were available for the BS and ANG populations, respectively. We utilized deregressed estimated breeding values (dEBVs) calculated based on the approach proposed by Garrick et al. [36] as pseudo-phenotypes. We used the GCTA software [37] based on the mixed linear model-based association analysis (MLMA) [38] following the model:

$${{\varvec{y}}}_{{\varvec{i}}}={\varvec{\mu}}+{\varvec{b}}{{\varvec{g}}}_{{\varvec{i}}}+{{\varvec{u}}}_{{\varvec{i}}}+{{\varvec{e}}}_{{\varvec{i}}}$$

where **y** is a vector of phenotypes; **µ** is the population mean, **g**_**i**_ is the number of copies of the reference allele, **b** is the allele substitution effect of the SNP, **u**_**i**_ is the polygenic effect of the i^th^ individual considered to be normally distributed **N**(0, **Gσ**^**2**^_**g**_), where **σ**^**2**^_**g**_ is the polygenic genetic variance and **G** is the genomic relationship matrix as previously described; and **e**_ij_ is a vector of residual effects with **e** ~ **N**(0, **Iσ**^**2**^_**e**_). We applied the Bonferroni method to correct for multiple testing. The significance threshold (*p* < 0.05) considered the effective population size (Ne = 80, Brito et al. [39]), the average length of a chromosome and the number of chromosomes (2n = 29) at the chromosome wide level [40, 41]. This correction method accounts for multiple testing and adjusts the significance thresholds of the SNPs as demonstrated by Goddard et al. [41].

### Gene annotation and functional analyses

To annotate the SNP associated with juniper consumption, we utilized the GALLO package [42] with a genomic window of 100 Kb upstream and downstream of the significant SNPs. The identified genes from the gene annotation were then used for functional analyses using the DAVID tool [43]. These analyses enabled the identification of the biological processes, molecular functions, cellular components, and pathways in which these genes are involved.

## Results

### Descriptive statistics

The descriptive statistics for juniper consumption, categorized by breed and sex, are presented in Table 2. In the ANG population, sex had a significant influence on juniper consumption, with males consuming more than females.

**Table 2:** Descriptive statistics for percentage of juniper consumption by breed and sex

*Breed*	*Sex*	*N*	*Min*	*Max*	*Mean*	*SD*
Boer x Spanish	F	4,027	-19.30	75.70	22.24	17.14
Boer x Spanish	M	2,424	-19.80	74.40	22.56	20.86
Angora	F	2,949	-23.60	36.00	4.65	10.18
Angora	M	1,127	-15.50	36.30	10.82	11.57

*N* Number of observations

*Min* Minimum percentage of juniper consumption observed

*Max* Maximum percentage of juniper consumption observed

*Mean* Average of juniper consumption

*SD* Standard deviation

The average consumption of juniper per sex, and line for BS and ANG is presented in Table 3. On average, juniper consumption increased by 105.8% for females from 2005 to 2017 and 127.5% for males from 2006 to 2017 in BS animals selected for high consumption of juniper (HIGH; Table 3). Also, for the lines selected for low consumption of juniper (LOW), there was a decrease in the consumption of 61.6% in females from 2005 to 2017 and 23.9% in males from 2006 to 2017 (Table 3). However, line was not recorded for all ANG animals, especially from 2010 to 2017. This gap hinders a comprehensive analysis of juniper consumption trends in ANG. The Additional file 1: Table S1 present the average consumption of juniper per year, sex, and line.

**Table 3:** The average consumption of juniper per goat, sex, and line

Breed	Sex	Year	Line	N	Min	Max	Mean
*Boer x Spanish*	F	High	1951	-15.00	75.70	28.73	16.95
	F	Low	2076	-19.30	67.90	17.56	14.33
	M	High	1185	-16.40	74.40	30.32	19.95
	M	Low	1239	-19.80	66.90	15.24	19.23
*Angora*	F	High	401	-21.50	58.50	10.62	7.87
	F	Low	304	-31.30	44.10	5.55	7.85
	M	High	93	2.90	50.00	23.17	8.66
	M	Low	24	2.50	44.00	19.89	6.64

*N* Number of observations

*Min* Minimum percentage of juniper consumption observed

*Max* Maximum percentage of juniper consumption observed

*Mean* Average of juniper consumption

*SD* Standard deviation

### Variance components and genetic parameters

The variance components and genetic parameters for juniper consumption are provided in Table 4. The heritability of juniper consumption was 0.43 ± 0.02 and 0.19 ± 0.03 for BS and ANG populations, respectively.

**Table 4:** Variance components and genetic parameters estimated for juniper consumption in Boer x Spanish composite and Angora goat populations

	**Boer x Spanish**		**Angora**	
	**Mean**	**SD**	**Mean**	**SD**
**h**^**2**^	0.43	0.02	0.19	0.03
High	0.22	0.05	-	-
Low	0.22	0.04	-	-
**Realized h**^**2**^	0.34	0.06^a^	0.13	0.03^a^
High	0.13	0.02^a^	-	-
Low	0.19	0.03^a^	-	-
**pe**	0.03	0.01	0.09	0.02
High	0.14	0.04	-	-
Low	0.15	0.04	-	-
**σ**^**2**^_**g**_	35.80	2.37	9.40	1.39
High	16.26	3.59	-	-
Low	15.63	3.33	-	-
**σ**^**2**^_**pe**_	2.12	1.08	4.41	1.16
High	9.56	2.24	-	-
Low	9.95	2.29	-	-
**σ**^**2**^_**e**_	45.80	0.98	35.00	0.93
High	45.43	1.43	-	-
Low	40.72	1.22	-	-
**t**	0.45	0.02	0.28	0.02
High	0.36	0.02	-	-
Low	0.38	0.02	-	-
**r**_**g**_	-0.04	0.11	-0.31	0.20

*h*^*2*^ Heritability

*HIGH* Selected line for high consumption of juniper

*LOW* Selected line for low consume of juniper

*pe* ratio of permanent environment

*σ*^*2*^_*g*_ genetic variance

*σ*^*2*^_*pe*_ permanent environment variance

*σ*^*2*^_*e*_ residual variance

*t* repeatability

*r*_*g*_ genetic correlation between juniper consumption and weaning weight

^a^standard error of the realized heritability

The repeatability for juniper consumption was 0.45 ± 0.02 and 0.28 ± 0.02 in the BS and ANG populations, respectively. With respect to the genetic correlation with weaning weight, a negative and weak genetic correlation (-0.04 ± 0.11) was observed in the BS population, while the correlation was moderate and negative (-0.31 ± 0.20) in the ANG population. When calculating variance components for each line separately for the BS population (line information was not available for all ANG animals), the heritability estimates decreased from 0.43 to 0.22 for each line (Table 4). The realized heritability estimates are 0.34 ± 0.06 and 0.13 ± 0.03 for BS and ANG, respectively. The variance components and genetic parameters for weaning weight are presented in the Additional file 2: Table S2.

### Genome-wide association studies results

**Fig. 1:**
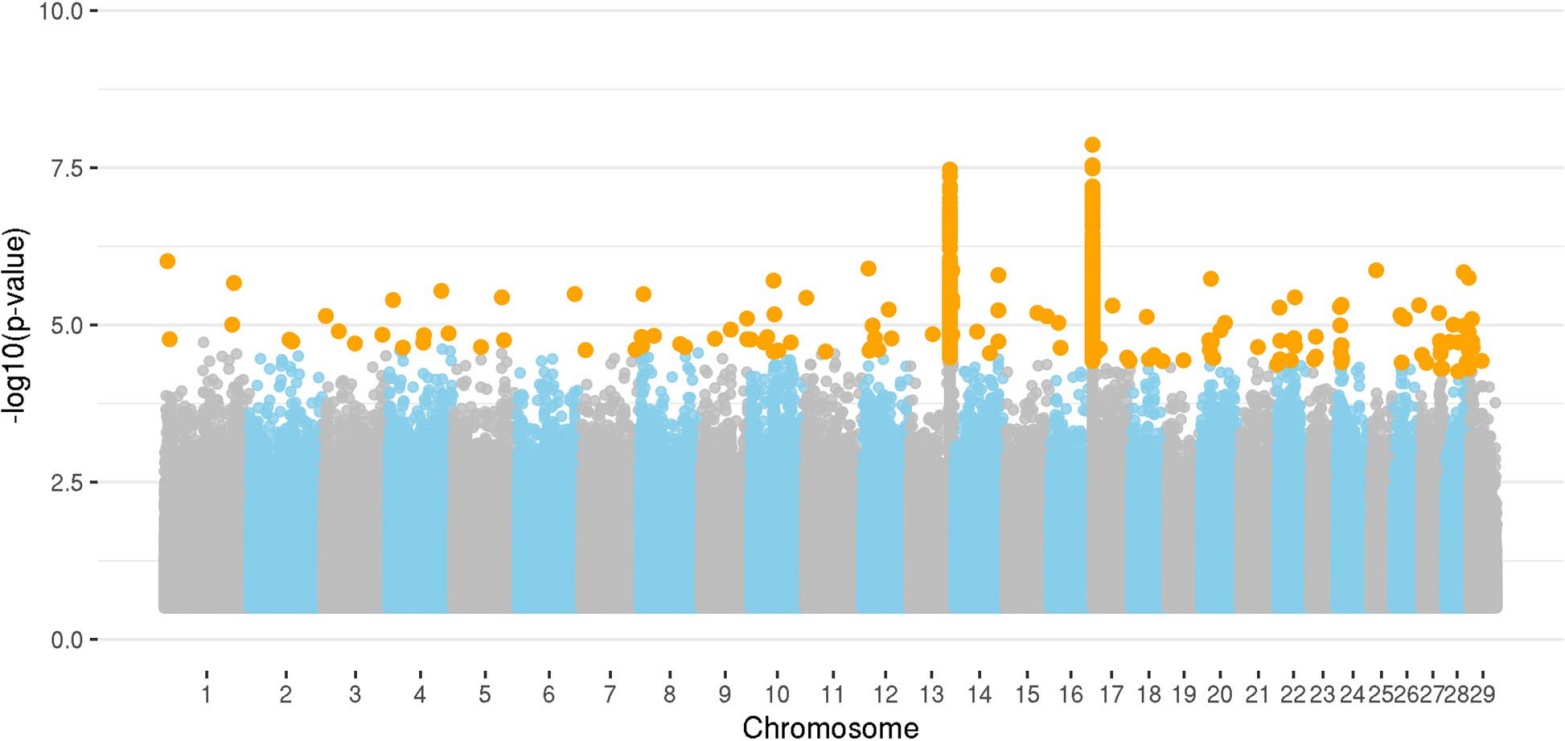
Manhattan plot for juniper consumption in a crossbred Boer x Spanish goat population based on whole-genome sequence data. Legend: Orange dots indicate the significant markers for juniper consumption

Figure 1 displays the Manhattan plot illustrating the GWAS analysis of juniper consumption in the BS population. A total of 571 significant SNPs were distributed across all autosomal chromosomes that exhibited a significant effect on juniper consumption. The two most significant peaks are located on the *Capra hircus* chromosome 13 (CHI13: 75,272,313–79,825,590) and CHI17 (327,164–378,403). These SNPs are associated with 231 positional genes, including 168 protein-coding genes, 34 long intergenic non-coding RNAs, 11 small nuclear RNAs, five immunoglobulin V genes, seven small nucleolar RNAs, three microRNAs, one miscellaneous RNA, and one processed pseudogene. A detailed description of the genes and their classifications is presented in Additional File 3: Table S3.

For the main genomic peaks identified, there are six genes located on CHI13 (*ENSCHIG00000002030, ENSCHIG00000002361, ENSCHIG00000002371, ENSCHIG00000005214, EYA2, ZMYND8*), and 26 genes on CHI17 (*ENSCHIG00000003453, ENSCHIG00000003521, LOC102174962, ENSCHIG00000008572, ENSCHIG00000009334, ENSCHIG00000011400, ENSCHIG00000017461, ENSCHIG00000017632, ENSCHIG00000020716, ENSCHIG00000024061, ENSCHIG00000025831, ENSCHIG00000023467, ENSCHIG00000011805, ENSCHIG00000020629, ENSCHIG00000010666, ENSCHIG00000011513, ENSCHIG00000015496, LOC108637841, KSR2, FICD, SART3, ENSCHIG00000003166, ENSCHIG00000005872, ENSCHIG00000006007, SH3D19, FHIP1A*). Notably, five genes located on CHI3 and CHI10 (*LOC102185841, LOC102190566, LOC102186401, LOC108636907, LOC102186483*) are associated with the olfactory receptor system.

Table 5 provides an overview of the functional analyses for the positional genes linked to juniper consumption within the BS population. The genes identified play a role in seven biological processes, eight molecular functions, three cellular components, and three pathways that are specifically associated with juniper consumption, including intestinal absorption (GO:0050892).

**Table 5:** Functional analyses conducted on the genes linked to juniper consumption in the Boer x Spanish goat population

	**Term**	**N genes**	***p*-value**	**Genes**
Biological Process				
GO:0010634	Positive regulation of epithelial cell migration	3	0.010	*ENSCHIG00000011473, ENSCHIG00000026002, ENSCHIG00000022660*
GO:0007179	Transforming growth factor beta receptor signaling pathway	4	0.010	*ENSCHIG00000007488, ENSCHIG00000011473, ENSCHIG00000022660, ENSCHIG00000017970*
GO:0009411	Response to UV	3	0.016	*ENSCHIG00000011503, ENSCHIG00000016647, ENSCHIG00000025781*
GO:0006909	Phagocytosis	3	0.024	*ENSCHIG00000019616, ENSCHIG00000013665, ENSCHIG00000014924*
GO:0050892	Intestinal absorption	2	0.058	*ENSCHIG00000009946, ENSCHIG00000019616*
GO:0043066	Negative regulation of apoptotic process	5	0.079	*ENSCHIG00000004678, ENSCHIG00000014974, ENSCHIG00000025831, ENSCHIG00000026002*
GO:0010468	Regulation of gene expression	3	0.090	*ENSCHIG00000007488, ENSCHIG00000021385, ENSCHIG00000011473*
*Molecular Function*				
GO:0051959	Dynein light intermediate chain binding	3	0.005	*ENSCHIG00000017543, ENSCHIG00000021643, ENSCHIG00000022612*
GO:0045505	Dynein intermediate chain binding	3	0.006	*ENSCHIG00000017543, ENSCHIG00000021643, ENSCHIG00000022612*
GO:0008569	ATP-dependent microtubule motor activity, minus-end-directed	3	0.008	*ENSCHIG00000017543, ENSCHIG00000021643, ENSCHIG00000022612*
GO:0005267	Potassium channel activity	3	0.011	*ENSCHIG00000003466*
GO:0005524	ATP binding	19	0.013	*ENSCHIG00000014297, ENSCHIG00000017543, ENSCHIG00000023716, ENSCHIG00000008083, ENSCHIG00000016664, ENSCHIG00000021643, ENSCHIG00000016963, ENSCHIG00000015887, ENSCHIG00000022612, ENSCHIG00000014954, ENSCHIG00000020551, ENSCHIG00000024773, ENSCHIG00000019938, ENSCHIG00000025781, ENSCHIG00000005472, ENSCHIG00000012319, ENSCHIG00000004524, ENSCHIG00000012697, ENSCHIG00000011473*
GO:0046872	Metal ion binding	19	0.021	*ENSCHIG00000014650, ENSCHIG00000017941, ENSCHIG00000014974, ENSCHIG00000022976, ENSCHIG00000024542, ENSCHIG00000024773, ENSCHIG00000021135, ENSCHIG00000025203, ENSCHIG00000020183, ENSCHIG00000025440, ENSCHIG00000025781, ENSCHIG00000012697, ENSCHIG00000017295, ENSCHIG00000019075, ENSCHIG00000004901, ENSCHIG00000010780, ENSCHIG00000011473, ENSCHIG00000019650, ENSCHIG00000010382*
GO:0004197	Cysteine-type endopeptidase activity	3	0.029	*ENSCHIG00000018930, ENSCHIG00000011503, ENSCHIG00000026002*
GO:0005539	Glycosaminoglycan binding	2	0.058	*ENSCHIG00000011473, ENSCHIG00000019067*
Cellular Component				
GO:0005886	Plasma membrane	24	0.059	*ENSCHIG00000017543, ENSCHIG00000019327, ENSCHIG00000014078, ENSCHIG00000022504, ENSCHIG00000020318, ENSCHIG00000024417, ENSCHIG00000021187, ENSCHIG00000015813, ENSCHIG00000014802, ENSCHIG00000020690, ENSCHIG00000003793, ENSCHIG00000026073, ENSCHIG00000004678, ENSCHIG00000011139, ENSCHIG00000004524, ENSCHIG00000000113, ENSCHIG00000012697, ENSCHIG00000013522, ENSCHIG00000017295, ENSCHIG00000011473, ENSCHIG00000017970, ENSCHIG00000013154, ENSCHIG00000016363, ENSCHIG00000000529*
GO:0035869	Ciliary transition zone	2	0.084	*ENSCHIG00000000494, ENSCHIG00000006328*
GO:0016021	Integral component of membrane	45	0.088	*ENSCHIG00000023329, ENSCHIG00000019327, ENSCHIG00000014078, ENSCHIG00000019622, ENSCHIG00000009140, ENSCHIG00000014974, ENSCHIG00000021187, ENSCHIG00000024773, ENSCHIG00000014717, ENSCHIG00000005144, ENSCHIG00000025440, ENSCHIG00000020690, ENSCHIG00000003793, ENSCHIG00000013959, ENSCHIG00000000464, ENSCHIG00000002685, ENSCHIG00000011354, ENSCHIG00000019075, ENSCHIG00000017791, ENSCHIG00000011473, ENSCHIG00000013154, ENSCHIG00000018122, ENSCHIG00000016363, ENSCHIG00000022504, ENSCHIG00000008083, ENSCHIG00000016664, ENSCHIG00000018308, ENSCHIG00000025118, ENSCHIG00000015813, ENSCHIG00000024762, ENSCHIG00000014802, ENSCHIG00000021652, ENSCHIG00000024122, ENSCHIG00000007488, ENSCHIG00000026073, ENSCHIG00000000255, ENSCHIG00000003466, ENSCHIG00000012753, ENSCHIG00000013522, ENSCHIG00000012697, ENSCHIG00000015461*
Pathways				
chx04724	Glutamatergic synapse	4	0.042	*ENSCHIG00000024417, ENSCHIG00000015813, ENSCHIG00000009680, ENSCHIG00000020690*
chx04024	cAMP signaling pathway	5	0.083	*ENSCHIG00000008083, ENSCHIG00000016664, ENSCHIG00000021423, ENSCHIG00000019792, ENSCHIG00000017295*
chx04014	Ras signaling pathway	5	0.084	*ENSCHIG00000023716, ENSCHIG00000012319, ENSCHIG00000019842, ENSCHIG00000021423, ENSCHIG00000024417*

**Fig. 2:**
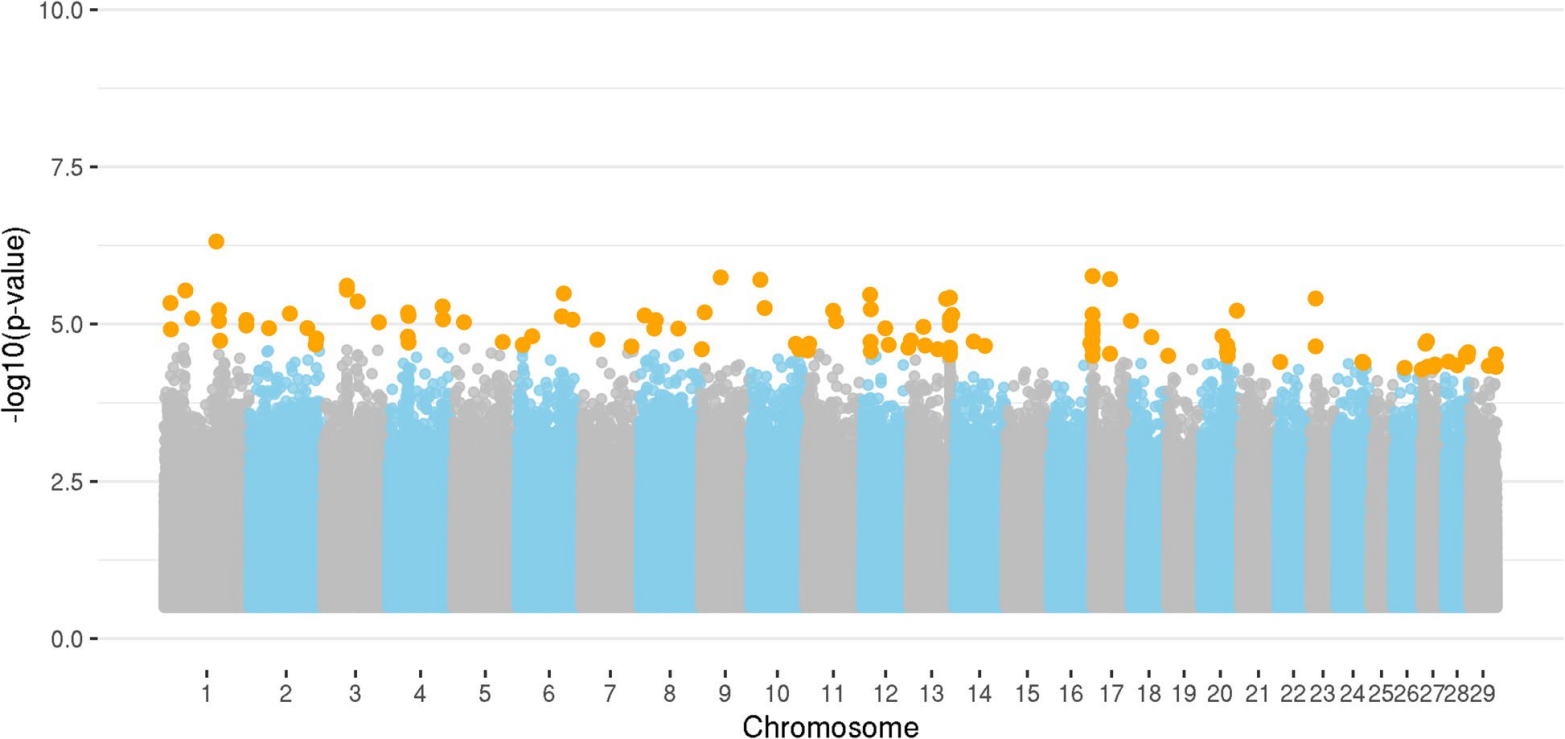
Manhattan plot for juniper consumption in an Angora goat population based on whole-genome sequence data. Legend: Orange dots indicate the significant markers for juniper consumption

Figure 2 presents the Manhattan plot for juniper consumption in the ANG population. A total of 116 SNPs were identified to have a significant impact (*p* < 0.05) on juniper consumption. These SNPs are associated with 183 genes, consisting of 138 protein-coding genes, 22 long intergenic non-coding RNAs, seven small nuclear RNAs, four immunoglobulin germline genes, three microRNAs, three processed pseudogenes, two pseudogenes, two ribosomal RNAs, and two small nucleolar RNAs. Similar genomic regions were identified for both populations. The CHI13 region contains 14 genes (*ENSCHIG00000001274, ENSCHIG00000002347, ENSCHIG00000002357, ENSCHIG00000003660, ENSCHIG00000005214, ENSCHIG00000009331, ENSCHIG00000010780, ENSCHIP00000012347, GINS1, ANKEF1, ITGA8, PCMTD2, ZMYND8, FZD8*), and the one on CHI17 contains 14 genes associated with juniper consumption (*ENSCHIG00000003453, ENSCHIG00000003521, ENSCHIG00000004635, ENSCHIG00000008572, ENSCHIG00000008793, ENSCHIG00000009334, ENSCHIG00000011400, ENSCHIG00000011805, ENSCHIG00000017461, ENSCHIG00000017632, ENSCHIG00000020716, ENSCHIG00000023467, ENSCHIG00000024061, ENSCHIG00000025831*). A detailed description of all the position genes along with their respective classifications is presented in Additional File 4: Table S4.

The gene *OR2Z1*, which is related to the olfactory receptor, has been annotated within the genomic regions where the SNPs exhibit a significant impact on juniper consumption. Table 6 presents the gene ontology terms associated with the genes identified in the ANG population regarding juniper consumption. The genes identified are significantly involved in four biological processes, six molecular functions, two cellular components, and nine metabolic pathways associated with juniper consumption.

**Table 6:** Functional analysis conducted based on the genes linked to juniper consumption in the Angora goat population

	**Term**	**N genes**	***p*-value**	**Genes**
**Biological Process**				
GO:0002376	Immune system process	3	0.005	*ENSCHIG00000001274, ENSCHIG00000014141*
GO:0007160	Cell–matrix adhesion	3	0.029	*ENSCHIG00000020502, ENSCHIG00000017512, ENSCHIG00000019633*
GO:0008206	Bile acid metabolic process	2	0.057	*ENSCHIG00000020709, ENSCHIG00000000506*
GO:0045596	Negative regulation of cell differentiation	2	0.096	*ENSCHIG00000025831*
**Molecular Function**				
GO:0004222	Metalloendopeptidase activity	6	0.000	*ENSCHIG00000020502, ENSCHIG00000025021, ENSCHIG00000015652, ENSCHIG00000019633, ENSCHIG00000015701, ENSCHIG00000001217*
GO:0005524	ATP binding	14	0.052	*ENSCHIG00000014981, ENSCHIG00000016126, ENSCHIG00000023289, ENSCHIG00000014206, ENSCHIG00000015009, ENSCHIG00000014879, ENSCHIG00000007275, ENSCHIG00000008522, ENSCHIG00000003216, ENSCHIG00000013314, ENSCHIG00000011751, ENSCHIG00000016690, ENSCHIG00000012374, ENSCHIG00000013396*
GO:0042626	ATPase activity, coupled to transmembrane movement of substances	2	0.068	*ENSCHIG00000003216, ENSCHIG00000025227*
GO:0046914	Transition metal ion binding	2	0.078	*ENSCHIG00000021789, ENSCHIG00000013424*
GO:0008017	Microtubule binding	4	0.083	*ENSCHIG00000008522, ENSCHIG00000010675, ENSCHIG00000011751, ENSCHIG00000005959*
GO:0004707	MAP kinase activity	2	0.084	*ENSCHIG00000012374, ENSCHIG00000013396*
**Cellular Components**				
GO:0045177	Apical part of cell	3	0.040	*ENSCHIG00000025789, ENSCHIG00000000147, ENSCHIG00000018661*
GO:0005764	Lysosome	4	0.073	*ENSCHIG00000018060, ENSCHIG00000009558, ENSCHIG00000013798, ENSCHIG00000005959*
**Metabolic Pathways**				
chx01523	Antifolate resistance	5	0.000	*ENSCHIG00000014981, ENSCHIG00000016126, ENSCHIG00000013314, ENSCHIG00000014206, ENSCHIG00000016690*
chx04976	Bile secretion	6	0.000	*ENSCHIG00000002450, ENSCHIG00000014981, ENSCHIG00000016126, ENSCHIG00000013314, ENSCHIG00000016690, ENSCHIG00000022594*
chx04657	IL-17 signaling pathway	5	0.002	*ENSCHIG00000021789, ENSCHIG00000013424, ENSCHIG00000014206, ENSCHIG00000012374, ENSCHIG00000013396*
chx02010	ABC transporters	4	0.006	*ENSCHIG00000014981, ENSCHIG00000016126, ENSCHIG00000013314, ENSCHIG00000016690*
chx04071	Sphingolipid signaling pathway	4	0.031	*ENSCHIG00000015908, ENSCHIG00000020626, ENSCHIG00000009558, ENSCHIG00000012374*
chx04024	cAMP signaling pathway	5	0.046	*ENSCHIG00000014981, ENSCHIG00000016126, ENSCHIG00000013314, ENSCHIG00000016690, ENSCHIG00000012374*
chx05418	Fluid shear stress and atherosclerosis	4	0.049	*ENSCHIG00000000147, ENSCHIG00000014206, ENSCHIG00000025722, ENSCHIG00000012374*
chx01240	Biosynthesis of cofactors	4	0.065	*ENSCHIG00000002450, ENSCHIG00000025722, ENSCHIG00000022594, ENSCHIG00000007275*
chx00830	Retinol metabolism	3	0.083	*ENSCHIG00000002450, ENSCHIG00000023075, ENSCHIG00000022594*

## Discussion

The objective of this study was to investigate the genetic background of predicted juniper consumption by BS and ANG goats in a divergently-selected population. In terms of measuring juniper consumption, it is important to interpret the results in the context of an interval scale. In the field of measurement, an interval scale implies that values can be ordered systematically, and the differences between them hold meaning and remain consistent across the entire range of measurements, even without an absolute zero point [17]. When interpreting the results, the values represent the percentage of juniper in their diets. On average, the composition of juniper consumed in the diets was 22.4% and 7.01% in BS and ANG diets, respectively.

Juniper consumption differences were observed in the ANG population regarding sex. In this population, males consumed more juniper than females (223%). Previous research on the effect of sex on fNIR predictions of juniper consumption reported that, when on the same diet, males had a 50% higher predicted percentage of juniper in the diet compared to females, which may account for some of the difference between populations [17]. Although not statistically comparable as the animals were not kept in the same pastures, numerically the BS population consumed more juniper than ANG, and previous studies reported similar findings [15, 44] that the composite animals [45, 46] might be more effective biological agents for juniper control. During droughts, pastoralists are reluctant to reduce their livestock numbers partly because they believe their animals are uniquely suited to the environment and management system they were raised in and believe this adaptiveness is genetic and environmental [47, 48]. These results provide a potential validation for this belief.

As observed in Table 3 and Additional File 1: Table S1, there has been a consistent improvement in juniper consumption by the lines selected for high consumption over the years. This improvement can be attributed to the selection that was applied in the populations for this trait since 2003 [49] and the positive results of the selection process. Over the course of nearly 15 years, animals were carefully chosen based on their estimated breeding values (EBVs) to accentuate the desired phenotypes while effectively managing the inbreeding coefficient to stay below 5%. This finding is consistent with previous studies and reinforces the idea that juniper consumption has varying levels of genetic control [18, 50]. This implies that the trait can be actively selected and exhibit significant improvements across generations. In this study, animals from both lines within population were first analyzed together. This was done because there was an exchange of breeding animals across lines and the lines were selected during a limited number of generations. Furthermore, the animals included in this study originated from a single farm and they might not represent the genetic background of the studied trait in the breeds. However, the results obtained do indicate that the trait is under genetic control and one can expect the same pattern in other goat populations. To confirm this hypothesis, future studies in other goat populations should be conducted to evaluate the genetic background of juniper consumption in more representative populations. We also calculated variance components per line for the BS population and observed heritability estimates of 0.22 for both HIGH and LOW lines. The realized heritability estimates also indicate that juniper consumption is heritable in both goat populations.

When considering weaning weight, the consumption of juniper showed a weak to moderate negative genetic correlation for both BS and ANG populations. In the case of BS, this implies a slight, almost negligible trend for genetic variants influencing juniper consumption to be linked with weaning weight. Conversely, for ANG, a moderate and negative association was observed. This suggests that does that consumed more juniper weaned lighter kids resulting in a lower EBV for weaning weight. Nonetheless, existing evidence suggests that animals that begin consuming juniper immediately after weaning tend to exhibit increased juniper consumption over time [51].

The identification of numerous SNPs and genes associated with juniper consumption indicates that the consumption of juniper is influenced by many genes with small effects. While the exact mechanism enabling goats to tolerate juniper consumption has not been fully established [14, 52], and the impact on the animals’ physiological systems can be quite variable. Goats are usually not significantly affected by the odor of juniper plants [14, 53]. In this study, five genes associated with olfactory receptor activity were identified for the BS population (*LOC102185841, LOC102190566, LOC102186401, LOC108636907, LOC102186483*), and one for ANG (*OR2Z1*). The identification of these genes could provide insights into the molecular mechanisms underlying the high juniper consuming goats’ ability to perceive and tolerate the consumption of juniper. Goats have a complex nasal cavity and presumably a high sensitivity to odors [54], which may allow them to detect the amount of plant secondary metabolites such as condensed tannins, mono- and sesquiterpenes in juniper and select plants that have lower levels of these defensive chemicals. Goats prefer *J. ashei* over *J. pinchotii* [44] and the former has 30 – 50% less volatile oils than the latter [44, 55], and browsed *J. ashei* has 60% less volatile oils than unbrowsed plants [11].

Another effect of juniper consumption is associated with the negative consequences that arise from exposure to high levels of monoterpenes [4]. Upon ingestion of juniper and subsequent passage through the rumen, plant secondary metabolites, particularly monoterpenoids, can be rapidly and easily absorbed through the intestinal wall without interacting with digestive enzymes due to their physicochemical properties [56]. In the BS population, the biological process of intestinal absorption (GO:0050892) was found to be influenced by leptin (*ENSCHIG00000019616—LEP*) and glucosaminyl (N-acetyl) transferase 3 (*ENSCHIG00000009946—GCNT3*). When camphor was intra-ruminally dosed, total serum camphor was five times lower in HIGH goats compared to LOW goats [57] and when fed a diet with a constant amount of monoterpenes, HIGH animals had a higher concentration of monoterpenes in their feces than LOW [58]. The leptin gene is known to affect feed intake and energy homeostasis, playing a crucial role in nutrient absorption [59]. Interestingly, when it comes to plant-derived molecular components, the presence of terpenoids enhances leptin sensitivity [60], leading to a decrease in animal consumption. Regarding glucosaminyl (N-acetyl) transferase 3, it is a gene that belongs to the N-acetylglucosaminyltransferase family. It plays a role in reducing the expression of catabolic genes involved in glucosamine metabolism [61]. Moreover, it is associated with exerting an anti-inflammatory effect on intestinal epithelial cells [62], and in liver damage [63]. These findings suggest that the genes possess mechanisms that enhance the tolerance of juniper components, thereby mitigating the negative effects of its consumption.

For the peak observed on CHI13 (75,272,313–79,825,590), most of the genes are long intergenic non-coding RNAs and small nucleolar RNAs. These genes have the potential to regulate neighboring genes, suggesting their involvement in enhancer-like activity [64, 65]. In the BS population, the identified genomic regions in this peak harbor the *EYA2* and *ZMYND8* genes, which play crucial roles in cell maintenance and regulation, contributing to growth and development processes [66, 67]. In the ANG population, although the significant genomic region was not as pronounced as in the BS population, several other genes including *GINS1*, *ANKEF1*, *ITGA8*, *PCMTD2*, *ZMYND8*, and *FZD8* were found within the region. These genes are involved in cellular maintenance and activities such as replication [68] and cell recovery [66, 67].

For BS, the genomic region located on CHI17 (327,164–378,403) harbors a small nucleolar RNA and two small nuclear RNAs, which play important roles in gene regulation [65]. Additionally, five immunoglobulin V genes overlap with this region, contributing to the differential capacity of generating an immune response to restore normal activities after intoxication [69]. There was also one processed pseudogene identified, which may function as a promoter for neighboring genes [70]. Some of the genes within this genomic region are uncharacterized proteins specific to the goat species. The remaining genes are protein-coding, with some yet to be fully recognized. Notably, the *KSR2* gene appears to be linked to consumption. *KSR2* is involved in multiple signaling pathways and plays a role in energy homeostasis and insulin resistance [71]. There are studies reporting that the consumption of terpenoids can be beneficial for health, promoting a therapeutic potential for insulin resistance and hyperglycemia [72]. In the ANG population, the region associated with this peak was primarily characterized by four immunoglobulin V genes and one processed pseudogene. Furthermore, we identified nine protein-coding genes, all of which have not been annotated yet. More extensive investigation is required to uncover the specific functions of these genes and their implications in the response to juniper consumption.

Notably, our findings underscore a relationship between juniper consumption and the gene ontology related to bile acid metabolic processes, as well as bile secretion metabolic pathways. A function of bile is to facilitate the elimination of toxic substances from the organism [73]. This observation implies that these metabolic processes could potentially contribute to the preference for juniper consumption in these particular genetic lines of goats.

Although not evaluated in this study, it is important to consider other factors that contribute to goats' ability to consume juniper. One such factor is the rumen microbiome, which plays a crucial role in degrading, deactivating, and detoxifying plant metabolites [74] Microbial activity can aid in the consumption of plants like juniper. Additionally, a significant proportion of goats is raised in harsh environmental conditions, where plants accumulate secondary metabolites as a defense mechanism against herbivores, heat, and water stress [75]. This suggests that the interaction between the microbiome, the animal, and the environment may contribute to the adaptability of goats to tolerate the negative effects of juniper consumption and being able to act as a controlling agent.

Another physiological aspect that may contribute to goats’ ability to handle the negative effects of juniper consumption is their high capacity to absorb less and excrete metabolites more efficiently compared to other species [76]. When components of juniper that were not utilized by rumen microorganisms reach the intestine, they continue the digestion process. These components are then absorbed, and any toxic compounds are processed by the liver, where they are metabolized, detoxified, and excreted [77]. Numerous mechanisms involving enzymes and proteins, some of which are related to the genes associated with juniper prediction, are involved in this process. Although some of these mechanisms are yet to be fully characterized, they appear to be strongly linked to juniper consumption. While this study offers significant insights into the genetic factors influencing juniper consumption, it is important to address certain limitations and challenges that were encountered during the study’s execution. Despite the utilization of a highly accurate predictive technique to analyze animal consumption patterns, its predictions might not be 100% precise. This potential variability could introduce bias, particularly in cases where the dataset size is limited. Another notable consideration is the gap in our knowledge concerning the animals’ weights during the experiment. Regrettably, our dataset lacked information regarding the animals’ weights when collecting fecal samples. This aspect represents an intriguing avenue for further investigation, as it could potentially shed light on the relationship between consumption patterns and animal development. The inclusion of weight data during the fecal sample collection phase could enhance the comprehensiveness of our findings. To compensate for the absence of weight data, we relied on information related to weaning weight, a trait that exhibits a strong correlation with weight in different ages [78]. While this alternative offered valuable insights, it is important to recognize that direct weight measurements during the experiment would have provided a more comprehensive perspective on how juniper consumption impacts animal development. Addressing this limitation in future studies could contribute to a more holistic understanding of the relationships involved.

## Conclusions

There were numerical differences in juniper consumption between BS and ANG goats, with BS exhibiting higher juniper consumption. Juniper consumption is heritable and can be increased through selective breeding. Various SNPs and candidate genes associated with juniper consumption were identified in both populations. The analyses revealed a potential connection between juniper consumption and genes associated with olfactory receptors. Furthermore, genes implicated in crucial processes such as intestinal absorption, including *Leptin* and *glucosaminyl (N-acetyl) transferase,* as well as those involved in bile secretion and bile metabolic processes, were also identified. Multiple uncharacterized genes were found to be related to juniper consumption, which opens opportunities for further genome annotation studies. Overall, these findings highlight the potential for genetic selection to change dietary preferences in goats, resulting in animals that are better adapted to their environment for more sustainable production and rangeland restoration.

### Supplementary Information

**Additional file 1. **

**Additional file 2. **

**Additional file 3. **

**Additional file 4. **

## Data Availability

All the data needed for the interpretation of the results are presented in the paper and in its supplementary materials. The raw datasets can be made available for research based upon reasonable requests (Dr. Luiz Brito; E-mail: britol@purdue.edu).
